# Clinical Implications of Mutations at Reverse Transcriptase Codon 135 on Response to NNRTI-Based Therapy

**DOI:** 10.2174/1874357900701010008

**Published:** 2007-08-20

**Authors:** Harout K Tossonian, Jesse D Raffa, Jason Grebely, Mark Viljoen, Annabel Mead, Milan Khara, Mark McLean, Ashok Krishnamurthy, Stanley DeVlaming, Brian Conway

**Affiliations:** 1 Department of Anesthesiology, Pharmacology and Therapeutics, University of British Columbia, Vancouver, Canada; 2 Department of Statistics and Actuarial Science, University of Waterloo, Waterloo, Canada; 3 Pender Community Health Centre, Vancouver Coastal Health, Vancouver, Canada

## Abstract

To evaluate the impact of mutations at reverse transcriptase codon 135 on treatment outcomes in patients receiving NNRTI-based antiretroviral therapy, a total of 68 patients (30 with and 38 without baseline mutations at codon 135) were evaluated. Median increases in CD4 counts were 135 and 90 cells/mm^3^ (p=0.32), virologic suppression (HIV RNA < 400 copies/mL) was achieved in 16 (53%) and 16 (42%) patients (p=0.50), while NNRTI resistance was detected in 10/14 (71%) and 16/22 (73%) in patients with and without mutations at codon 135, respectively. Patients who experienced a virologic breakthrough and had a baseline mutation at codon 135 were more likely to evolve a single NNRTI resistance mutation (8/14 *vs* 4/22, p=0.029) but less likely to evolve multiple NNRTI resistance mutations (2/14 *vs* 12/22, p = 0.033). Mutations at codon 135 do not affect response rates, but affect the pattern of development of NNRTI resistance mutations. This has important implications for the subsequent use of newer NNRTIs such as etravirine in salvage therapy.

## INTRODUCTION

The use of highly active antiretroviral therapy (HAART) has proven remarkably effective in controlling the progression of HIV/AIDS and prolonging survival, but these benefits can be compromised by the development of drug resistance. Recent estimates of the prevalence of drug resistance during the first years of widespread use of potent antiretroviral therapy have indicated that about 70% of treated adults with detectable viremia have isolates with drug resistance mutations [1, 2]. Clinically significant resistance to some drugs, notably non-nucleoside reverse transcriptase inhibitors (NNRTIs), can emerge after just a brief exposure to this class of medications. A single point mutation, such as the K103N, is sufficient to cause high-level NNRTI resistance to all currently available agents in this class [3]. The majority of NNRTI resistance can be accounted for by the presence of recognized mutations in the reverse transcriptase (RT) gene. However, certain additional polymorphisms present in different settings may result in decreased drug susceptibility. In individuals with such polymorphisms who were never treated with NNRTIs, phenotypic analysis has revealed evidence of measurable decreases in drug susceptibility [4].

A number of reports have suggested that mutations at RT codon 135 (I to one of T/M/V/L/R/K) may impact NNRTI susceptibility. *In vitro*, a 2.5-fold increase in IC_50_ to nevirapine or delavirdine [5] and to nevirapine or efavirenz [6] was observed in the absence of any prior drug exposure. Some databases list the I135T mutation as being associated with NNRTI resistance [7]. More recently, genetic changes at codon 135 were associated with the subsequent accumulation of mutations in subjects receiving an NNRTI-containing regimen, indicating that such mutations may provide an alternate route for the development of high-grade NNRTI drug resistance [8]. Other reports; however, have not identified such a relationship [9].

Over the past several years we have developed a successful program for the treatment of HIV-infected injection drug users (IDUs) within a directly observed therapy (DOT) program using simplified treatment regimens [10, 11]. In our IDU drug naive population, we have detected mutations at RT codon 135 (mainly 135T) in more than 40% of cases [12]. These mutations occurring at such a high frequency may have a significant impact on response rates to NNRTIs and their use in clinical practice. With this in mind, we sought to evaluate the CD4 cell counts and plasma viral load responses to NNRTI-based regimens as well as the evolution of nucleoside reverse transcriptase inhibitor (NRTI) and NNRTI resistance mutations in patients with or without mutations at codon 135 prior to the initiation of therapy.

## MATERIALS AND METHODOLOGY

HIV-infected IDUs with pre- and post-treatment genotypic resistance testing who received NNRTIs for the first time and for more than 1 month were included in this retrospective study. HAART regimens were received through a DOT program at the Pender Community Health Centre, a multidisciplinary clinic located on the downtown east side of Vancouver, Canada. HAART regimens were individualized, based on considerations of efficacy and toxicity with regimens being mainly based on nevirapine or efavirenz along with two NRTIs.

CD4 cell counts and HIV plasma viral load responses to NNRTI-based therapy were compared in patients with or without mutations at RT codon 135 at baseline. HIV plasma viral load was measured using the Amplicor HIV-1 Monitor™ assay (Roche Diagnostics, Mississauga, ON). Plasma viral load was defined as below the limit of quantitation if it was <400 HIV RNA copies/mL. Immunologic response was monitored using the CD4 cell count, measured by flow cytometry at the local reference laboratory. HIV plasma viral load and CD4 cell counts were measured at baseline, and at approximately three month intervals or more frequently if clinically indicated. Efficacy was evaluated based on the most recent HIV plasma viral load and CD4 cell count measurements or the last one prior to a change in therapy if applicable. Baseline data were also collected on patient demographics, antiretroviral treatment history and the presence of any pre-treatment drug resistance mutations, as defined by the International AIDS Society guidelines (IAS-USA table, October 2006) [13].

To evaluate the evolution of NRTI and NNRTI resistance mutations in the setting of virologic breakthrough, genotypic drug resistance testing was done using the VirtualPhenotype™ Assay, VIRCO Lab, Mechelen, Belgium. Genotypic resistance tests were performed at baseline and at the time of each confirmed virologic breakthrough (viral load >400 copies/mL). In cases where genotypic testing was not done previously, frozen plasma samples were available for retrospective testing. The rates of accumulation of individual and multiple NRTI and NNRTI resistance mutations were calculated in patients failing NNRTI-based therapy. In addition, the rates of acquisition of NRTI and NNRTI resistance mutations were compared in patients with and without mutation 135 at baseline.

Tests for independence between two discrete variables were done using the χ^2^ or Fisher’s exact test, as appropriate. Continuous variables were assessed using either Student’s t-test (age) or the Mann-Whitney test (absolute and change from baseline in CD4 cell count). All reported p-values were two-sided, and p-values below a significance level of 0.05 were considered statistically significant.

## RESULTS

The study included a total of 68 patients identified from a retrospectively collected database of IDUs who received NNRTIs for the first time and for more than 1 month and who had pre- and post-treatment genotypic resistance testing. At baseline, 30 patients (19 male) were identified as having a mutation at RT codon I135 [135T (17), 135V (7), 135M (3), 135R (2), 135L (1)] while 38 patients (25 male) had no mutations at codon 135. All patients carried subtype B virus. Median baseline CD4 cell counts and plasma viral loads were 190 cells/mm^3^, 81,350 copies/mL and 190 cells/mm^3^ (p-value=0.99), 65,800 copies/mL (p-value=0.44) in the two groups, respectively. Twenty-three patients received nevirapine, 6 received efavirenz and 1 received delavirdine in the first group, while 32 patients received nevirapine, 5 received efavirenz and 1 patient received delavirdine in the second group. In all cases, the agent of interest was given along with 2 NRTIs and/or protease inhibitors to constitute a HAART regimen. The NRTIs commonly used included lamivudine (n=25 and 32) and didanosine (n=24 and 32) in patients with and without baseline mutations at codon 135, respectively. Other NRTIs, including stavudine, zidovudine and tenofovir, were used as part of HAART in the remaining few patients with equal distribution in the two study groups. PIs were used in 7 regimens only including lopinavir, sequinavir, nelfinavir and indinavir. The baseline characteristics of study patients are presented in Table 1.

**Fig. (1):**
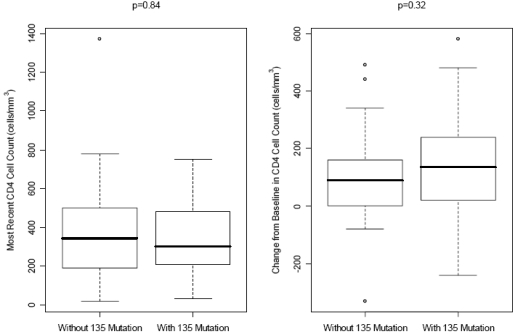
Median CD4 cell counts and median increases in CD4 cell counts at the latest follow-up visit in patients with and without baseline mutations at codon 135.

**Fig. (2):**
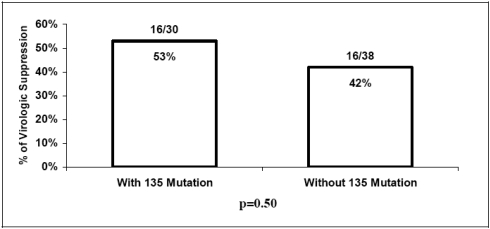
Virologic suppression at the latest follow-up visit in patient with and without baseline mutations at codon 135.

At a median follow-up period of 15 months, the median changes in CD4 cell counts were +135 cells/mm^3^ and +90 cells/mm^3^ (p=0.32) while the CD4 cell counts were 300 cells/mm^3^ and 345 cells/mm^3^ (p=0.84) in patients with and without baseline mutations at codon 135, respectively (Fig. 1). Virologic suppression (HIV RNA <400 copies/mL) was achieved in 16/30 (53%) of patients having mutations at codon 135 at baseline and 16/38 (42%) in patients without baseline mutations at codon 135 (p=0.50) (Fig. 2).

As seen in Table 2, of those not suppressed with a baseline codon 135 mutation, 10/14 (71%) had NNRTI resistance mutations [K103N (6), Y181C (3), G190A (2), V108I (1)] and 4/14 (29%) had NRTI resistance [M184I/V (3), L74V (2), thymidine analogue mutations (TAMs) (2)]. In patients with no such mutation, 16/22 (73%) had NNRTI resistance [K103N (10), Y181C (9), G190A (7), V108I (2), L100I (1), V106M (1), Y188C (1)] and 12/22 (55%) had NRTI resistance [M184I/V (12), L74V (3), TAMs (1)] (p > 0.05 for all comparisons). In patients who experienced a virologic breakthrough and had a baseline mutation at codon 135, 8/14 (57%) evolved a single NNRTI resistance mutation, a finding that was observed in only 4/22 (18%) patients who did not have such a baseline mutation (p = 0.029). The latter group was more likely to evolve multiple mutations (12/22 [55%] cases), a finding that was much less frequently observed (2/12 [14%] cases) in the setting of a 135 mutation at baseline (p = 0.033).

## DISCUSSION AND CONCLUSION

The virologic impact of mutations at codon 135 is not clear despite the fact that such mutations are extremely common. It is likely that mutations at RT codon 135 affect NNRTI susceptibility by virtue of their proximity, in the p51 component of the RT dimer, to the NNRTI binding site [14]. The β7-β8-loop (residues 132-140) in the p51 subunit of HIV RT contributes to the formation of the base of NNRTI-binding pocket. In one study, mutations at codons 132, 135 and 138 in the 51 subunit of RT conferred high-level resistance to nevirapine and delavirdine and low level resistance to efavirenz [15]. Position 135 of HIV RT is known as the anchor position of the HLA-B51 restricted cytotoxic T lymphocyte (CTL) responses [16]. CTL escape mutations occur at critical sites within HLA-restricted CTL epitopes where an amino acid substitution may abrogate epitope-HLA binding, reduce T-cell receptor recognition, or generate antagonistic CTL responses [17]. According to Mallal *et al.*, a strong association was observed between HLA-B51 and the presence of mutations at codon 135 of RT. In particular, all individuals with HLA-B51 had an I to T (79%) or V (21%) mutation at position 135 suggesting that non-synonymous mutations at position 135 of RT may lead to viral escape from host CTL responses [18]. Other studies; however, have associated HLA-B51 with slow progression to AIDS [19]. Thus, the explanation for the differences in the number of mutations observed in our study may be secondary to differential representation of MHC alleles in both study groups. We plan to undertake the appropriate evaluations in our cohort to determine whether MHC variations provide a unifying explanation for our observations.

Mutations at RT codon 135 are not widely recognized as associated with more classical NNRTI-resistance mutations and their clinical implications are unclear. Brown *et al.* found that the amino acid site 135 of RT was associated with reduced susceptibility to both nevirapine and delavirdine [5]. Vavro *et al.* reported that a decreased susceptibility to efavirenz and nevirapine at baseline was seen in viruses with mutations at codon 135 in antiretroviral naïve patients [8]. In addition, they showed that mutations at codon 135 at baseline were associated with the accumulation of NNRTI-resistance mutations [8]. Harrigan *et al.*; however, found no relationship between the presence of mutations at codon 135 and virologic response [9]. In agreement with previous reports, we did not detect significant differences in immunologic or virologic responses in patients on NNRTI-based HAART as a function of the baseline genotype at codon 135. Further, such mutations at baseline were not significantly associated with the development or accumulation of NNRTI mutations while on therapy in the setting of loss of virologic suppression. Nevertheless, if resistance occurred, patients who had baseline 135 mutations were more likely to evolve single NNRTI resistance mutations (8/14 *vs* 4/22, p=0.029) which is in agreement with results from previous studies [8]. However, patients who had baseline 135 mutations were less likely to evolve multiple NNRTI resistance mutations (2/14 *vs* 12/22, p=0.033) and possibly some NRTI mutations such as the M184V (3/14 *vs* 12/22, p=0.08). This contrasts with previous reports which suggested that the presence of baseline mutations at codon 135 may accelerate the accumulation of more classical NNRTI resistance mutations. It should be emphasized that much of these data were generated with non-subtype B strains of HIV. This may explain why, in populations infected with subtype B virus, very different results may be obtained [8, 20].

Although it is difficult to attribute all the mutations we observed to NNRTI treatment, it is generally agreed that NRTIs do not affect the evolution of NNRTI-associated mutations or mutations selected by non-reverse transcriptase inhibitors. However, one recent publication reports some intriguing associations between NNRTI and NRTI mutations. Cane *et al.* identified 24 accessory RT codon mutations, including mutations at codon 135, as significantly associated with the accumulation of TAMs [21]. In our study, we did not find a significant association between mutations at codon 135 and TAMs, however, most of our patients were not on HAART regimens containing stavudine or zidovudine nor did they have complex resistance patterns including TAMs. Outside of this context, we are not aware of any reports of NRTIs selecting for or associated with mutations at codon 135. In the absence of any evidence to the contrary, we must conclude that the patterns of genetic change we have observed are associated with NNRTI use.

One important implication of our results relates to the possible sequencing of NNRTIs in clinical practice, with the availability of second generation agents in this class. It may be that in patients with baseline mutations at codon 135, NNRTI resistance may be associated with the development of a single additional mutation, increasing the likelihood of agents such as etravirine remaining effective. This would not be the case if multiple NNRTI mutations were to develop, a situation that may be more likely with subtype B virus wild type at the 135 codon of RT.

The study has several limitations. First, it was conducted in a relatively small number of patients. Second, the study was done on IDUs enrolled in a DOT program and thus, the results might not be generalized to non-IDU populations or non-DOT settings. Third, resistance mutations considered where only those based on the IAS-USA guidelines and some other crucial or unknown mutations might not have been properly evaluated including minor mutations such as L283I. According to Brown *et al.*, this can lead to further decrease in susceptibility to NNRTIs in the context of a pre-existing 135 mutation [5]. Fourth, our study did not account for the effect of the different amino acid positions at codon 135 as some specific mutations might be more important than others. Fifth, phenotypic assays were not done, without which it is hard to know surely whether the observed mutations had any effect on the sensitivity or resistance to NNRTIs. Historically, however, and in the bulk virtual phenotypes performed in our centre, mutations at codon 135 were associated with a 1.2-1.3-fold decrease in NNRTI susceptibility, suggesting a minimal effect on the efficacy of agents in this class as a result of this mutation alone.

In conclusion, our results indicate no significant differences in CD4 cell counts and HIV plasma viral load responses to NNRTI-based regimens as a function of baseline 135 genotype. However, in patients with baseline mutations at codon 135 and experiencing virologic breakthrough, there were more evolution of single and less evolution of multiple NNRTI resistance mutations. This may have important implications with respect to the initial selection of patients to receive NNRTI-based therapy at baseline with a view to sequencing NNRTIs using newer agents in this class in subsequent courses of therapy.

## Figures and Tables

**Table 1.** Baseline Patient Characteristics

	With Baseline 135 Mutation	Without Baseline 135 Mutation	p-Value
N	30	38	
Gender Male (%) Female (%)	19 (63%) 11 (37%)	25 (66%) 13 (34%)	0.99
Age (years) Mean SD	45.7 9.4	39.2 7.1	0.003
CD4 (cells/mm^3^) Median Q1 – Q3	190 130 - 270	190 110 - 270	0.99
Viral load (copies/mL) Median Q1 – Q3	81,350 32,700 - >100,000	65,800 26,780 - >100,000	0.44
Follow-up period (months) Median Q1 – Q3	15.7 5.0-37.2	14.2 5.4-21.6	0.50
Regimen based on Nevirapine (%) Efavirenz (%) Delavirdine (%)	23 (77%) 6 (20%) 1 (3%)	32 (84%) 5 (13%) 1 (3%)	0.76
RT mutations (%)	3 (10%)	3 (8%)	0.99
Naïve to antiretrovirals (%)	14 (47%)	21 (55%)	0.63

**Note:** Q1 indicates first quartile; Q3 third interquartile; RT indicates reverse transcriptase; SD indicates standard deviation.

**Table 2:** Rates of Drug Resistance Following Virologic Breakthrough on NNRTI-Based Therapy

Mutations	With Baseline 135 Mutation	Without Baseline 135 Mutation	p-Value
NRTIs L74V M184I/V TAMs 1 Mutation >1 Mutation	4/14 (29%) 2 (14%) 3 (21%) 2 (14%) 1 (7%) 3 (21%)	12/22 (55%) 3 (14%) 12 (55%) 1 (5%) 7 (32%) 5 (23%)	0.18 0.99 0.08 0.55 0.12 0.99
NNRTIs L100I K103N V106M V108I Y181C Y188C G190A 1 Mutation >1 Mutation	10/14 (71%) 0 (0%) 6 (43%) 0 (0%) 1 (7%) 3 (21%) 0 (0%) 2 (14%) 8 (57%) 2 (14%)	16/22 (73%) 1 (5%) 10 (45%) 1 (5%) 2 (9%) 9 (41%) 1 (5%) 7 (32%) 4 (18%) 12 (55%)	0.99 0.99 0.99 0.99 0.99 0.29 0.99 0.43 0.029 0.033
No resistance mutations	4/14 (29%)	5/22 (23%)	0.71

**Note:** NNRTI indicates non-nucleoside reverse transcriptase inhibitor, NRTI indicates nucleoside reverse transcriptase inhibitor, TAM indicates thymidine analogue mutation.
